# Mycotoxins in Norwegian food and feed

**DOI:** 10.1186/1751-0147-54-S1-S6

**Published:** 2012-02-24

**Authors:** Gunnar Sundstøl Eriksen, Per Erik Clasen, Ellen Christensen

**Affiliations:** 1 Norwegian Veterinary Institute, P.O 750 Sentrum, N-0106 Oslo, Norway

## 

Mycotoxins are toxic secondary fungal metabolites. A large variety of mycotoxins have been described, but only a minority of them occur regularly in feed and food items under normal conditions and pose a risk to feed and food safety.

The important moulds producing toxins during storage belong to the genera of *Penicillium*, or A*spergillus.* The most important storage toxins are probably ochratoxin A and aflatoxins. The growth of moulds and toxin production after harvest are dependent on temperature and humidity and, consequently, the most effective mitigation strategy is to maintain the commodities dry and at a low temperature during storage.

Aflatoxin is normally not produced in the Norwegian climate and the occurrence is restricted to imported feed and food. Ochratoxin A is produced by *Penicillium spp* and *Aspergillus spp*. Occurrence of ochratoxin A in selected commodities is included in the national food and feed surveillance programs. Only low concentrations of ochratoxin A have been found in compound feed as well as in feed ingredients [1-5]. It is concluded that the procedures for handling of feed seem to be sufficient to limit the production of ochratoxin A.

Elevated levels of ochratoxin A are occasionally reported from dried food items, including dried fruits and coffee beans.

The fungi producing aflatoxin are generally restricted to a tropical climate. Consequently, aflatoxins in Norwegian feed and food are mainly limited to imported feed, food and ingredients. The main aflatoxin sources in feed have been maize and maize-derived feed ingredients, but the levels in compound feed have been low [1-5]. Aflatoxins do not constitute a high risk to animal health nor to human health through transfer to humans through consumption of animal-derived food products in Norway. The toxin occurs in imported dried food items such as dried figs and nuts, but the results from the present control regime shows that the risk to human health due to consumption of aflatoxin-contaminated foods is limited.

## Penitrems

Penitrems are neurotoxic mycotoxins produced by *Penicillium spp*. Penitrems have generally been reported from visibly damaged commodities, and reported intoxications from humans are limited. Dogs, however, may ingest mouldy commodities such as mouldy food or feed, food waste or apples lying on the ground. Ingestions of such items have been reported to result in severe intoxications with a variety of clinical symptoms characteristic for neurotoxic poisoning, including shivering, tremors, and tachycardia [6,7].

## Trichotecenes

Trichothecenes are a large family of related mycotoxins, but only deoxynivalenol (DON), and T-2 and HT-2 toxins are frequently found in Norwegian grains. Trichothecenes have been analysed in grains sampled at delivery for many years. The levels of DON in Norwegian grains may be increasing. In addition, the toxins have also been analysed in processed feed. Other trichothecenes such as nivalenol (NIV) are also found in Norwegian grains, but both frequency of positive samples and the concentrations reported are generally low [1-5]. The levels are, with few exceptions, below the maximum limits for feed ingredients. According to EFSA, DON in feed may affect feed intake of pigs at levels from 0.35 – 0.9 mg/kg feed [8]. The levels of DON in pig feed are therefore close to those that may have a negative impact on feed intake. Other species are less sensitive, and no effects on other species are expected at the current levels in feed. The apparent increasing trend of DON in Norwegian grains is also of concern.

## Ergot

Ergot refers to fungi of the genus *Claviceps*, where *C*. *purpurea* is the most prominent. Presumptive intoxications of moose and roe deer have been reported from different parts of Norway. Three moose were found dead, while the remaining moose and the roe dear were all suffering from typical signs of ergotism, like convulsive and gangrenous symptoms [9]. Fungal sclerotia have later been collected from grass in the pasture in the area and ergotamine and analogues have been identified and described [10]. These toxins are not analysed for in processed feed. The fungus is common in rye, but the toxin-containing sclerotia are removed during processing at the mills. Traces have been reported from wheat and barley in other countries and may be present even in Norway. The levels have not been regarded as any significant risk to animal health or welfare.

## Conclusions

Trichothecenes and DON in particular, are the main trichothecenes of concern in processed feed. DON occurs in processed feed in concentrations close to levels found to reduce the feed intake and feed conversion in pigs. The neurotoxic mycotoxin penitrem A is of concern for animals consuming moldy feed, and in particular for dogs ingesting food waste and rotten and molded apples. Grazing wild-life, such as moose and roe deer, may be intoxicated by ergot.
